# Stability of Individuals’ Definitions of Success and the Influence of Perceived Motivational Climate: A Longitudinal Perspective

**DOI:** 10.3389/fpsyg.2020.01326

**Published:** 2020-06-11

**Authors:** Christina G. L. Nerstad, Robert Buch, Anders Dysvik, Reidar Säfvenbom

**Affiliations:** ^1^Faculty of Social Sciences, Oslo Business School, OsloMet—Oslo Metropolitan University, Oslo, Norway; ^2^Department of Leadership and Organizational Behavior, BI Norwegian Business School, Oslo, Norway; ^3^School of Communication, Leadership and Marketing, Kristiania University College, Oslo, Norway; ^4^Department of Physical Education, Norwegian School of Sport Sciences, Oslo, Norway

**Keywords:** goal orientation, perceived motivational climate, stability, longitudinal design, cadets

## Abstract

In the present study, we investigated the stability and malleability of cadets’ definitions of success (mastery and performance goal orientations) contextualized within a certain motivational climate (mastery and performance climates). Based on data from three military academies, the results revealed that cadets’ goal orientations and their perceptions of the motivational climate remained relatively stable throughout the 2 years of study across three time-points. We also found that a mastery climate predicted individual mastery orientation, and that a performance climate predicted individual performance orientation. These findings contribute to achievement goal theory by clarifying the importance of considering goal orientation contextualized within a certain motivational climate over time. Implications for future research and practice are discussed.

## Introduction

A current reality in some military academies is that cadets’ differing levels of performance is publicly displayed. For instance, a recent ethnographic study of 56 cadets attending a Norwegian military academy, the cadets were ranked based on their skills and performance; the best performers were lined up to the left and the poor performers to the right (Magnussen and Boe, 2017). Such ranking and competition was by some cadets experienced as particularly destructive for their consequent performance. The study findings clearly illustrate how military academy contexts (i.e., motivational climate) valuing performance rather than mastery goals may result in detrimental outcomes. On the other hand, if military training contexts focus on mastery goals, it may facilitate positive outcomes such as group cohesion (Le Foll et al., 2019). Another interesting and important question is whether the perceived motivational climate influence individual’s achievement goal orientations over time. This is the focus for the present study.

According to achievement goal theory (AGT) individuals have different goals or purposes for engaging in achievement behavior (Ames, 1984). These goals represent a framework for how individuals approach and react when in an achievement situation (e.g., military academies) as well as the meaning that they assign to the achievement context (Baranik et al., 2007; Harwood et al., 2008). Individuals are predisposed to act in manners referred to as achievement goal orientations (Roberts et al., 2007). Goal orientations concern how individuals define success by validating their ability based on a self-referenced standard (i.e., mastery orientation) or relative to the ability of an other-referenced standard (i.e., performance orientation) (Nicholls, 1989; Vandewalle et al., 2019). Goal orientations have been found to play an important role in determining individuals’ attributions, attitudes, and performance (Payne et al., 2007; Jagacinski et al., 2010). Their importance to the mentioned outcomes has led some scholars to examine goal orientation stability because it may have implications for educational practice (e.g., military academy), in addition to provide a foundation for future intervention work (e.g., Fryer and Elliot, 2007; Payne et al., 2007; Jagacinski et al., 2010; Tuominen-Soini et al., 2011; Corker et al., 2013). For example, if goal orientation represents a stable trait, it can serve as a useful tool in identifying the way in which military academy cadets’ approach goals and should advise the development and delivery of training (Zweig and Webster, 2004). However, if goal orientation variability over time exists, such a finding has important implications for the salience of the design of the situational goal structure (Ames, 1992c). With respect to the extant literature on goal orientation stability and/or change (e.g., Fryer and Elliot, 2007), several limitations constrain the strengths of the contributions. First, most of the studies that have investigated goal orientation stability typically do not go beyond the length of a college semester, leaving some uncertainty regarding stability over longer time periods (Payne et al., 2007). A rare exception in this respect is the study by Corker et al. (2013) who found that over 4 years college, students’ goal orientations were not as stable as personality traits, meaning that some variability existed. These findings seem to undermine the total stability of goal orientation and emphasize the importance and need for a further determination of its stability over time (Payne et al., 2007). Accordingly, the i question regarding goal orientation stability and/or change—particularly in military academy contexts—remains unresolved (Payne et al., 2007; Corker et al., 2013; Magnussen and Boe, 2017).

Second—and just as important—the examined time periods have often been confounded with systematic changes in the learning environment (e.g., final exams), which may create either a stronger or weaker goal orientation situation (Payne et al., 2007; Corker et al., 2013). A more precise delineation between the context and individual characteristics seems warranted as individuals’ goal orientation has previously been suggested to represent a “somewhat stable individual difference factor that may be influenced by situational characteristics” (Button et al., 1996, p. 28). The extent to which situational characteristics actually influence goal orientation implies the need for precise measurement of different constructs and how they relate to each other over time. In a related vein, an important limitation of the existing goal orientation stability research is the lack of examining goal orientations contextualized within a certain climate (e.g., Corker et al., 2013; Becker et al., 2018). Although several scholars mention the relevance of climate (e.g., classroom goal structures) in influencing individuals goal orientation (e.g., Corker et al., 2013; Bardach et al., 2019), the extant research on goal orientation stability have, in large part, neglected to examine its influence. Therefore the long-term stability of goal orientation accounting for the impact of situational characteristics, such as the perceived motivational climate, remains unclear (Payne et al., 2007; Corker et al., 2013; Becker et al., 2018). The perceived psychological motivational climate (i.e., mastery and performance climates), defined as individuals perceptions of the existent criteria of success and failure in the situation, is likely to influence individual goal orientation to affect behavioral, affective, and cognitive outcomes (Chen and Mathieu, 2008; Nerstad et al., 2013a). The motivational climate is highly relevant because individual differences in goal orientations may be shaped by individual differences in perceived mastery and performance climate structures (Ames, 1992c; Nerstad et al., 2018a). In other words, it is assumed that dispositional goal orientation is triggered by perceived situational characteristics that energize a specific achievement behavior (Ames, 1992c; Bardach et al., 2019).

The main purpose of our study is to increase current knowledge of the stability/malleability of goal orientations by examining the relationship between the perceived motivational climate and goal orientation by way of a three-wave longitudinal study across 2 years. By more precisely delineating between situational influences in the form of the perceived motivational climate and goal orientation, we also test Ames (1992b,c) suggestion that mastery orientation should be fostered by long-term exposure to a perceived mastery climate, while a perceived performance climate should encourage the development of a performance orientation. We thereby intend to contribute to the AGT literature by clarifying the relevance of the perceived motivational climate in influencing individual goal orientation over time. Our aim is to answer the calls for additional research on the impact of the perceived motivational climate on individual goal orientation (Fryer and Elliot, 2007; Corker et al., 2013; Becker et al., 2018). We also extend previous goal orientation research by making contextual information more clear and testing Nicholls (1989) and Ames (1992c) situated AGT (cf., Payne et al., 2007). An understanding of the motivational climate in relation to individual goal orientation over time is relevant because it can have important implications for both achievements directed practice and future intervention work.

## Theory and Hypotheses

### Achievement Goal Theory

An important characteristic strength of AGT is that it incorporates both personal and situational motivational facets (Nicholls, 1989; Ames, 1992c; Nerstad et al., 2013b; Bardach et al., 2019). The personal facet is individual goal orientation, while the situational facet is represented by the motivational climate.

#### Individual Goal Orientation

Originally, Nicholls (1984) distinguished between two types of goal orientation that have been presented somewhat different in their conceptualization (task vs. ego orientations, performance vs. mastery orientations, or performance vs. learning orientations). Despite these differences, it has been argued that these conceptualizations share enough in common to justify convergence (e.g., Rawsthorne and Elliot, 1999). In line with Ames (1992c), we adopt the mastery- versus performance-orientation conceptualization for the present study.

According to traditional AGT, when mastery oriented, an individual defines success based on self-referenced ability, and success concerns mastery, skill development, and learning (Ames, 1992c; Campbell, 2006). A mastery orientation has been shown to predict higher academic performance as well as job performance above and beyond the Big Five and cognitive ability (Payne et al., 2007; Corker et al., 2013). It is further associated with lower state anxiety, greater inclination for feedback seeking, and greater learning (e.g., Payne et al., 2007; Lau and Nie, 2008).

On the other hand, when performance oriented, an individual’s perception of norm-referenced ability relative to others is the main criterion of defining success (Nicholls, 1984, 1989). Accordingly, individuals strive to demonstrate their capabilities (Campbell, 2006). Empirical findings have indicated both adaptive and maladaptive outcomes of performance orientation (Midgley et al., 2001; Payne et al., 2007). For example, higher levels of performance orientation have been found to predict academic success, although it also has been associated with higher state anxiety (Payne et al., 2007; Senko et al., 2011; Corker and Donenellan, 2012).

It should be noted that there exists more recent approaches to AGT, such as the hierarchical model of achievement motivation (Elliot, 1999; Elliot and Conroy, 2005) where goals exists in the form of approach or avoidance goals. This development of a trichotomous model including mastery, performance approach and performance avoidance goals was presented as an initial revision of traditional AGT (VandeWalle, 1997; Elliot and Dweck, 2005). A 2 × 2 achievement framework was later proposed incorporating mastery-avoidance goals (Elliot, 1999; Elliot and Dweck, 2005). These models propose that individuals strive to be competent or strive to avoid appearing incompetent, thereby making it possible to distinguish goals based on their valence or to what extent the outcome is pleasant or unpleasant (Roberts et al., 2007). Both the trichotomous (VandeWalle, 1997; Elliot and Murayama, 2008) and 2 × 2 (Elliot and Thrash, 2001) approaches to AGT present energizing constructs that are different from traditional AGT, which is a social cognitive theory (Roberts, 2012). Social cognitive theories of motivation typically examine how individuals cognitively process and develop their views on achievement under various social influences and contexts (Roberts, 1992; Bandura, 1997), thereby *not* assuming the satisfaction of needs to be the main determinant and energizing force of behavior oriented toward competence (Elliot and Dweck, 2005). According to Nicholls (1989), goal orientations concern the criteria that make individuals feel successful in a given situation, rather than how they define competence and the valence of striving, as assumed by the trichotomous and 2 × 2 approaches (Roberts, 2012). Therefore, instead of extending the traditional AGT, the hierarchical approach (i.e., trichotomous and 2 × 2) has been argued to represent a modernization of “the traditional motivation arguments by Atkinson and McClelland by introducing the competence arguments of Nicholls, not the other way around!” (Roberts, 2012, p. 34). Accordingly, since the focus of our study is on how individuals define success and what it takes to achieve success within a certain achievement setting (i.e., military academy), and because the hierarchical model does not take these aspects into account (Papaioannou et al., 2012), we chose to position our research in line with traditional AGT (Nicholls, 1989; Ames, 1992b, c).

#### The Stability of Goal Orientations

Given the empirical evidence for predictive validity for both goal orientation dimensions, scholars have pursued to clarify whether goal orientation is a stable individual difference variable (trait) or whether goal orientation variability exists (Vandewalle et al., 2019). Corker et al. (2013) meta-analyzed studies investigating stability and change in goal orientation during the college years. Their findings showed that college students mastery orientation decreased (average Cohen’s *d* = −0.39) over the course of a semester in the majority of studies. Performance orientation (average Cohen’s *d* = 0.00) did not change to a considerable degree. The existing evidence shows that mastery orientation typically decreases over the course of a semester, while a performance orientation does not seem to change (Corker et al., 2013). Goal orientation also appears to be less stable than the Big Five dimensions over the course of a semester (Corker et al., 2013). The authors further conducted a 4-year longitudinal study among college students where they found that the mastery orientation mean levels declined over time, while performance orientation mean levels remained constant. In terms of differential stability, the results indicated stability in both orientations, although the results supported the meta-analytic evidence by suggesting that stability coefficients were smaller for goal orientation compared to the Big Five personality traits. Accordingly, the term *disposition* (or individual difference) may best describe an individual’s characteristic goal orientation (Campbell, 2006; Vandewalle et al., 2019).

In concurrence, another relevant meta-analysis investigated goal orientation stability to clarify its importance for organizational interventions (e.g., training). The results indicated that over the short term, goal orientation was relative stable and comparable with those calculated for the Big Five attributions (Payne et al., 2007). Nevertheless, when the time interval was longer, the stability coefficient became weaker (Payne et al., 2007). In sum, these findings seem to undermine total goal orientation stability over the long run (beyond the length of a college semester). We therefore hypothesize the following:

Hypothesis 1: Goal orientations are relatively stable over time.

#### Relevance of the Perceived Motivational Climate for Goal Orientations

Although self-cognitions in the form of goal orientation are assumed to be relatively enduring, they are nonetheless dynamic and open to contextual influences such as the perceived motivational climate (Ames, 1992c; Papaioannou et al., 2012; Roberts, 2012; Vandewalle et al., 2019). The primary concern in achievement situations (e.g., military training, sport, or work), is the person’s perception of the competence needed to meet situational demands. The individual interprets the extant criteria of success and failure in the achievement context and perceives the behaviors necessary to achieve success and/or avoid failure (Roberts, 2012). Mastery and performance orientations are elicited by the perceived motivational climate, which consequently results in different motivational patterns (Ames, 1992c).

The motivational climate is identified as individuals’ perceptions of the existing criteria of success and failure emphasized through the policies, practices, and procedures in the achievement situation (cf., Nerstad et al., 2013a). Such a climate defines how individuals should be evaluated, the goals they ought to pursue, and how they should relate to tasks as well as to each other (Ames, 1984; Ames and Ames, 1984a, b). The motivational climate is represented by two basic goal reward structures: a mastery climate and a performance climate (Ames, 1992b, c). In a mastery climate, rewards tend to rely more on aspects such as progress, effort, self-improvement, and cooperation (Ames, 1984, 1992b). Individuals’ achievements are more positively independent of each other. The achievement process and success is viewed in light of a process of achieving mastery and learning or compared to what the individual has accomplished in the past (Ames, 1984; Nerstad et al., 2018b).

In contrast, a performance climate endorses an egoistic motivation in which social comparison information is highly salient (Nicholls, 1979). The important criterion is whether the person is a “winner” or a “loser” (Ames and Ames, 1984a). This illustrates a situation of negative interdependence among individuals, where the likelihood of one person achieving a goal or getting a reward is reduced by the presence of another more capable individual (Ames, 1984; Ames and Ames, 1984a; Černe et al., 2014). In a performance climate, individuals’ interest in comparing their own performance with that of significant others typically strengthens and reinforces them to demonstrate their ability (Ames and Ames, 1984a; Roberts et al., 2007).

A mastery orientation should emerge more frequently among individuals in a mastery climate. This because value is placed on the process of learning through emphasis on effort and the willingness to apply effort based learning strategies; self-referenced standards; opportunities for self-directed learning; meaningful learning; appropriate levels of challenge; realistic goal-setting; recognition of individuals effort; non-normative evaluation; task variety; opportunities for choice; and reasonable demands (Ames, 1992c). Further, when evaluation is private, informative, and focuses on personal progress and individual mastery, a mastery orientation is likely to be the outcome (Ames, 1992c).

Because Corker et al. (2013) found clear support for a decline in mastery orientation over time one might question what the possible explanation for such a deterioration might be. As argued by Corker et al. (2013), students may enter college with high levels of natural engagement and curiosity, which may disappear, particularly in large university classrooms. In such classrooms, pedagogical tactics may not be as engaging due to one-way communication or pure lecturing. Also, interpersonal comparisons are often explicitly or implicitly encouraged, and students may have little autonomy. This may facilitate a performance climate and consequently a drop in mastery orientation (Corker et al., 2013).

If the goal structure of military training is mastery involved from the first day of, it is likely that cadets will not experience a drop in and even an increase in individual mastery orientation. One explanation might be that they manage to continue focusing on the value and benefits of learning and skill development as an end in itself, and consequently, mastery orientation stability is to be expected (Ames, 1992c; Corker et al., 2013).

On the other hand, a performance goal is likely to emerge to a larger extent when cadets experience a performance climate. The above mentioned empirical findings suggesting that a performance orientation remained relatively stable across time may be explained by a rather stable classroom goal structure emphasizing performance involving criteria of success, i.e., being better than others or a competitive system of evaluation (Ames, 1992c). Even societal norms can play a salient role in shaping an educational system (Ames and Ames, 1984a). When teachers/leaders/coaches place substantial importance on being “number one,” being the “best,” getting A’s, or being in the top group, individuals performance orientation most likely will remain steady over time. In addition, Corker et al. (2013) argue that post-college rewards may be related to the importance of outperforming others in terms of, for example, competing for the best job opportunity. Such a perceived instrumentality may consequently contribute to maintaining performance goal mean levels over time (Corker et al., 2013). These arguments align with Ames (1992b,c) suggestion that mastery orientation should be adopted by long-term exposure to a perceived mastery climate, while a perceived performance climate should encourage the development of a performance orientation (Standage et al., 2003). We therefore hypothesize:

Hypothesis 2: There is a positive relationship between (a) perceived mastery climate and mastery orientation, and there is a negative relationship between (b) perceived performance climate and mastery orientation.
Hypothesis 3: There is a positive relationship between (a) perceived performance climate and performance orientation, and there is a negative relationship between (b) perceived mastery climate and performance orientation.

## Materials and Methods

### Sample and Procedure

Data for this study was collected from cadets in three Norwegian military academies at three points in time. Time 1 data was collected at the end of the participants first year at the academy. Time 2 data was collected at the end of the second year, and Time 3 data was collected at the end of participants’ third and final year at the academy. When responding to the survey, the participants were informed that the survey had been approved by the Norwegian Social Science Data Services (NSD), and their responses would be treated confidentially in order to reduce the presence of response distortion (Podsakoff et al., 2003). All cadets volunteered to participate and gave their written consent after receiving information about the study. They also received a personal code so that it was possible to match the data from each measurement occasion. A researcher connected to the research project was present at all academies to administer the questionnaires. The participating cadets received and completed the paper and pencil questionnaire in class. After completion, the cadets handed in the questionnaire during the same plenary session. Cadets who were not able to be present for the class session, were given the opportunity to complete the questionnaire at a later time.

To secure the highest possible response rate over time all respondents received an advance notice of the study. Topic salience was emphasized in the information letter which the respondents received. As noted above, the cadets received an identification number (code) to secure anonymity. Also, the questionnaires were distributed personally as meta analytic findings suggest that such a procedure leads to higher response rates (Anseel et al., 2010).

At Time 1, the initial sample included a total of 248 individuals (84% response rate). At Time 2, 167 (57% response rate) individuals responded to the survey. Finally, 161 (55% response rate) individuals completed the survey at Time 3. At the first measurement occasion (Time 1) the sample comprised 89.5% men and 10.5% women with a mean age of 23.6 years (*SD* = 2.63). Further, 72.8% of the cadets reported high school (general) as their educational level, while 15.6% reported civil university/college for the period of 1 to 3 years. The rest reported an educational level of either elementary school, civil university/college education for the period of 3 to 6 years, or civil university/college education for the period of 7 years or more. With respect to military academy affiliation, approximately 40% of the total sample represented Academy A, 36% represented Academy B, and 24% represented Academy C.

It should also be noted that with respect to age, military experience and rank, Norwegian military academy students are comparable to those of other NATO countries (Johansen et al., 2014). In general, individuals who attend military academies are characterized as healthy, self-sufficient, and a resilient group although they are exposed to a relatively authoritative, hierarchical, and competitive educational style (Buch et al., 2017). To address such contextual ambiguousness military academies were considered as interesting research units over time.

### Measures

All variables were measured at each measurement time point.

#### Goal Orientation

To measure goal orientation, we used the Perception of Success Questionnaire (POSQ; Roberts et al., 1998), which consists of six items measuring mastery orientation and six items measuring performance orientation. Sample items include “I feel most successful when I reach personal goals” (mastery orientation) and “I feel most successful when I am the best” (performance orientation). Respondents recorded their responses on a 5-point scale (1 = totally disagree to 5 = totally agree). The Cronbach’s α for a mastery orientation was 0.94 at Time 1, 0.95 at Time 2, and 0.91 at Time 3. For a performance orientation, the Cronbach’s α was 0.92 at Time 1, 0.92 at Time 2, and 0.92 at Time 3.

#### Perceived Motivational Climate

To measure individual perceptions of mastery and performance climate, we used the Norwegian version (Roberts and Ommundsen, 1996) of the Perception of Motivational Climate in Sport Questionnaire (PMCSQ; Seifriz et al., 1992). Sample items include “Trying hard is rewarded” (mastery climate) and “Cadets are punished for mistakes” (performance climate). Respondents recorded their responses on a 5-point scale (1 = totally disagree to 5 = totally agree). The Cronbach’s α for a perceived mastery climate was 0.82 at Time 1, 0.83 at Time 2, and 0.81 at Time 3. For a perceived performance climate, the Cronbach’s α was 0.79 at Time 1, 0.83 at Time 2, and 0.82 at Time 3.

#### Control Variables

In the present study, the three military academies were included. Because these academies (i.e., air, navy, and army) may have some notable differences in structure and course content, which can affect goal orientation, we controlled for academic affiliation (represented by three dummy variables). Finally, we controlled for age (measured at Time 1), gender (men = 0; women = 1), and prior educational level (where 1 represented “elementary school,” 2 represented “high school (general),” 3 represented “high school (vocational),” 4 represented “civil university/college for the period of 1 to 3 years,” 5 represented “civil university/college education for the period of 3 to 6 years,” and 6 represented “civil university/college education for the period of 7 years or more”) to rule them out as. alternative explanations for the observed findings.

#### Analytical Strategy

We analyzed the data in several steps. First, we performed a confirmatory factor analysis (CFA) with the use of the LISREL 8.80 program to determine whether the items reflected the constructs they were intended to measure. More specifically, following Kuvaas et al. (2012) we estimated a multiple indicator multiple cause (MIMIC) model to control for sample heterogeneity when performing the CFA (cf., Bollen, 1989; Muthén, 1989). Because “ordinal variables are not continuous and should not be treated as if they are” (Jöreskog, 2005, p. 10), we used the robust maximum likelihood estimator to accommodate the ordered categorical data. On the basis of recommendations in the literature (Pitts et al., 1996) for establishing construct validity in longitudinal measurements, the MIMIC-CFA was performed using the Time 1-measurements of mastery and performance goals. We applied common guidelines (e.g., the root mean square error of approximation [RMSEA] of <0.08, the comparative fit index [CFI] of >0.95, the non-normed fit index [NNFI], also referred to as the Tucker-Lewis index [TLI] of >0.95) to evaluate whether there was an acceptable model fit (Hu and Bentler, 1999; Marsh et al., 2005).

Second, to investigate our research questions using our longitudinal data, we followed recommendations in the literature (e.g., Singer and Willett, 2003; Hox, 2010) and performed longitudinal hierarchical linear modeling (HLM) with SPSS 19. In the present study, the data is hierarchical in the sense that the three measurement occasions are nested within individuals. The use of HLM and longitudinal data has several advantages. In contrast to a time-lagged research design in which each construct is measured once, longitudinal HLM incorporates development. More specifically, HLM allows us to estimate a trajectory of individual change in achievement goals and differentiate between concurrent levels of achievement goals and the change in achievement goals over time. Also, because cases that only consist of one or two measurements contribute less to the results of the longitudinal regression (Snijders and Bosker, 1999), differences among individuals with regards to the number of measurements (i.e., missing data) do not represent a problem (e.g., Hedeker and Gibbons, 1997; Hox, 2010). Finally, longitudinal data has more degrees of freedom. As a result, estimates obtained via HLM analysis are more efficient than those obtained in cross-sectional analysis (Wittekind et al., 2010).

Our model contained two levels of analysis where measurements over time represented level 1 and individuals represented level 2. Prior to the analysis, we set the most-frequent value in the categorical predictor gender to zero (Wittekind et al., 2010) and grand-mean centered the continuous predictors (Hofmann and Gavin, 1998). We coded time using consecutive numbers starting from zero in order to facilitate the interpretation of the intercept as the expected outcome on the first occasion (Hox, 2010).

## Results

### Preliminary Analyses

The MIMIC-CFA we performed on the full scales of a 4-factor model representing mastery and performance orientation and mastery and performance climate showed satisfactory fit indices (χ^2^ [576] = 955.35, *p* < 0.05; RMSEA = 0.047; CFI = 0.99; NNFI = 0.99). Furthermore, all factor loadings were statistically significant with a mean standardized loading of 0.82, which supports the convergent validity of the constructs (Anderson and Gerbing, 1988). Also, the scales displayed high internal consistency with reliability estimates ranging from α = 0.79 to α = 0.95 (Nunnally and Bernstein, 1994). We report descriptive statistics, correlations, and reliability estimates in Table 1.

**TABLE 1:** Descriptive statistics, scale reliabilities, and correlations.

	Mean	*SD*	1	2	3	4	5	6	7	8	**9**	**10**	**11**	**12**	**13**	**14**	**15**	**16**	**17**	
1. Academy A	0.40	0.49																		
2. Academy B	0.36	0.48	−0.61**																	
3. Academy C	0.24	0.43	−0.46**	−0.42**																
4. Gender	1.10	0.30	−0.16**	0.09	0.08															
5. Age T1	26.61	2.63	0.11	0.00	−0.13*	–0.06														
6. Prior education	2.50	0.90	–0.10	0.02	0.09	–0.11	0.48**													
7. Mastery climate T1	3.75	0.62	0.28**	−0.18**	–0.12	−0.19**	0.01	–0.02	**(0.82)**											
8. Mastery climate T2	3.68	0.65	0.43**	−0.29**	−0.18*	−0.20*	–0.05	–0.15	0.49**	**(0.83)**										
9. Mastery climate T3	3.68	0.57	0.36**	−0.31**	–0.09	–0.06	–0.08	–0.11	0.29**	0.49**	**(0.81)**									
10. Performance climate T1	3.08	0.61	0.14*	–0.12	–0.04	–0.08	0.02	0.06	–0.09	0.03	–0.04	**(0.79)**								
11. Performance climate T2	2.97	0.67	0.11	–0.05	–0.08	–0.11	–0.02	0.14	0.10	−0.19*	–0.05	0.50**	**(0.83)**							
12. Performance climate T3	3.03	0.66	0.17*	–0.08	–0.12	–0.10	–0.15	0.03	–0.08	–0.09	–0.04	0.56**	0.48**	**(0.82)**						
13. Mastery goals T1	4.17	0.87	0.21**	–0.10	−0.13*	0.02	–0.01	–0.11	0.45**	0.26**	0.20*	−0.15*	0.04	–0.03	**(0.94)**					
14. Mastery goals T2	4.15	0.95	0.28**	−0.16*	–0.16	–0.09	0.01	0.09	0.25**	0.34**	0.38**	–0.01	–0.10	–0.05	0.27**	**(0.95)**				
15. Mastery goals T3	4.28	0.72	–0.06	0.13	–0.07	0.03	–0.05	–0.02	0.05	0.11	0.14	–0.01	0.09	–0.05	0.16	0.22*	**(0.91)**			
16. Performance goals T1	3.03	0.96	0.07	–0.08	0.01	–0.08	−0.16*	–0.01	0.09	0.07	0.20*	0.32**	0.18*	0.20*	0.15*	0.19*	0.10	**(0.92)**		
17. Performance goals T2	2.89	0.95	0.01	0.07	–0.09	0.00	–0.15	–0.04	0.20*	0.06	0.20*	0.13	0.29**	0.27**	0.19*	0.14	0.16	0.48**	**(0.92)**	
18. Performance goals T3	2.97	0.93	0.04	0.06	–0.11	–0.07	−0.22*	–0.08	0.08	0.13	0.19*	0.26**	0.13	0.33**	0.12	0.17	0.14	0.53**	0.50**	**(0.92)**

Gender: Male = 1; female = 2. Time 1 = end of first year, Time 2 = end of second year, Time 3 = end of third year. Educational level: 1 = elementary school, 2 = high school (general), 3 = high school (vocational), 4 = civil university/college for the period of 1 to 3 years, 5 = civil university/college education for the period of 3 to 6 years, 6 = civil university/college education for the period of 7 years or more. Coefficients within parentheses and in bold are Cronbach’s alpha coefficients. *p < 0.05, **p < 0.01.

### Primary Analysis

To explore our hypotheses, we built four models (Hox, 2010) for each of the goal orientations. Model 0, which is the unconditional model (null model) contained only the level-1 intercept. In model 1, we included the predictor time as the level-1 slope to incorporate the longitudinal data structure. This allowed us to assess change in goal orientation over measurement occasions. In model 2, we entered the control variables. Finally, in model 3, we entered the time-varying predictors, mastery climate, and performance climate. Tables 2, 3 report the results of these models.

**TABLE 2:** Results of multilevel analysis.

	Mastery orientation				
	Model 0	Model 1	Model 2	Model 3	
Variables	Est.	Est.	Est.	Est.	Stand. Coeff.^b^
**Fixed effects**					
Intercept	4.17***	4.14***	4.00***	3.82***	
Time		0.05	0.07	0.10	0.09
**Control variables**					
Affiliation (1 = Academy A)			0.41**	0.27**	0.15**
Affiliation (1 = Academy B)			0.11	0.13	0.07
Gender (0 = Men, 1 = Women)			0.05	0.15	0.05
Age at Time 1			–0.01	–0.01	–0.04
Education			–0.00	0.03	0.03
**Time-varying predictors**					
Mastery climate				0.43***	0.31***
Performance climate				−0.13*	−0.11*
Variation within individuals	0.57***	0.58***	0.58***	0.57***	
Variation between individuals	0.19***	0.18**	0.12*	0.06	
Deviance (χ^2^)	1235.29	1234.19	1172.57	1118.85	
Decrease in deviance (Δχ^2^ ^a^)		1.10	61.62***	53.72***	

The full ML estimator was used to calculate this decrease in deviance (Δχ^2^) which can be considered a way of expressing effect size in multilevel modeling; we used the equation suggested by Hox (2010) to derive the standardized coefficients: Standardized coefficient = (unstandardized coefficient × standard deviation of the explanatory variable)/standard deviation of the outcome variable. *p < 0.05, **p < 0.01, ***p < 0.001.

**TABLE 3:** Results of multilevel analysis.

	Performance orientation				
	Model 0	Model 1	Model 2	Model 3	
Variables	Est.	Est.	Est.	Est.	Stand. Coeff.^b^
**Fixed effects**					
Intercept	2.95***	3.09***	4.50***	4.45***	
Time		–0.09	–0.02	0.03	0.03
**Control variables**					
Affiliation (1 = Academy A)			0.08	–0.02	–0.01
Affiliation (1 = Academy B)			0.02	–0.00	–0.00
Gender (0 = Men, 1 = Women)			–0.19	–0.15	–0.05
Age at Time 1			−0.06**	−0.05**	−0.17**
Education			0.03	0.03	0.03
**Time-varying predictors**					
Mastery climate				0.11	0.07
Performance climate				0.34***	0.25***
Variation within individuals	0.48***	0.47***	0.47***	0.46***	
Variation between individuals	0.44***	0.45***	0.41***	0.35***	
Deviance (χ^2^)	1279.62	1276.20	1225.31	1193.98	
Decrease in deviance (Δχ^2a^)		3.42	50.89***	31.33***	

The full ML estimator was used to calculate this decrease in deviance (Δχ^2^) which can be considered a way of expressing effect size in multilevel modeling; we used the equation suggested by Hox (2010) to derive the standardized coefficients: Standardized coefficient = (unstandardized coefficient × standard deviation of the explanatory variable)/standard deviation of the outcome variable. *p < 0.05, **p < 0.01, ***p < 0.001.

The null model for mastery orientation showed a between-person variance in mastery orientation of 0.19 (*p* < 0.01), and a within-person variance of 0.57 (*p* < 0.01) in mastery orientation over time. However, the non-significant fixed effect of time (γ = 0.05, *n.s.*) in model 1 indicated that no clear positive or negative trend over time exists with respect to mastery orientation. We therefore found support for Hypothesis 1, predicting relative stability in individual goal orientation over time.

Adding the control variables in model 2 improved model fit, as indicated by the statistically significant reduction in model deviance. The results indicated a significant positive relationship between Academy A affiliation and mastery orientation (γ = 0.41, *p* < 0.01). This indicates that the cadets at Academy A were more mastery oriented. The other control variables were not found to be significantly related to a mastery orientation (see Table 2). This indicates that age at Time 1, gender, and education did not represent alternative explanations for the observed findings.

Introducing the time-varying predictors of perceived mastery climate and perceived performance climate in model 3 led to further improvement in model fit (Δχ^2^ = 53.72, *p* < 0.001), demonstrating that model 3 best fit the data. In addition, from model 2 to model 3, variance decreased significantly, which is another indicator of the quality of the multilevel model (Singer and Willett, 2003). More specifically, in model 2, the within-person variance was 0.58, and the between-person variance was 0.12. Hence, the total variance was 0.70 and the decrease in total variance amounted to 10%. While the decrease in within-person variance was marginal (from 0.58 to 0.57), the between-person variance was reduced from 0.12 (*p* < 0.05) to 0.06 (*n.s.*), indicating that when controlling affiliation, gender, age, and education, the perceived motivational climate (mastery climate and performance climate) explains an additional 50% of the variance between individuals in mastery orientation. This reduction and significant fixed effects of mastery climate (γ = 0.43, *p* < 0.001) and performance climate (γ = −0.13, *p* < 0.05) provided support for Hypothesis 2, which stated that there is a positive relationship between (a) perceived mastery climate and mastery orientation, and that there is a negative relationship between (b) perceived performance climate and mastery orientation.

To test the relationships of perceived performance and mastery climate with performance orientation, we followed a similar procedure as described above. The null model for performance orientation (model 0), showed a within-person variance of 48 (*p* < 0.001) in performance orientation over time and a between-person variance performance orientation of 0.44 (*p* < 0.001). As with mastery orientation, introducing time as a linear predictor in model 1 did not significantly decrease model deviance, and the fixed effect of time was not significant, indicating that no clear positive or negative trend over time exists in the data. Adding the control variables in model 2, however, improved model fit, and the fixed effects of the time-invariant predictor age at Time 1 suggested that age differences between can explain between-person variance in performance orientation. The finding that age at Time 1 was significantly related to performance orientation (γ = −0.06, *p* < 0.01), suggests that the higher the age of the cadets the lower their performance orientation is. None of the other control variables were significantly related to a performance orientation (see Table 3), which may suggest that academic affiliation, gender, and education did not seem to represent alternative explanations for the observed findings. In model 3 we introduced the time-varying predictors, perceived performance climate, and perceived mastery climate. The introduction of these predictors resulted in a reduction in total variance of 13.6%, and a statistically significant decrease in model deviance (Δχ^2^ = 31.33, *p* < 0.01). In support of Hypothesis 3a, perceived performance climate (γ = 0.34, *p* < 0.001) was significantly related to performance orientation. Hypothesis 3b, however, was not supported as the fixed effect of perceived mastery climate (γ = 0.11, *n.s.*) failed to reach statistical significance (see Table 3).

## Discussion

The purpose of the present study was to longitudinally examine the relationship between individual differences in perceived motivational climate and goal orientation. Particularly, the study addresses how individuals perceive the success cues of the particular context (i.e., military academy) and then incorporate those perceptions into their goal orientations when striving toward success. Our findings reveal that (1) achievement goal orientations did not change in a predictable direction over time; and (2) the perceived motivational climate influences individual achievement goal orientation.

### Theoretical Implications

Although we observed significant within-person variation across measurement occasions, we did not find a clear positive or negative trend over time with respect to mastery or performance goal orientation across 2 years of military training. This suggests that although goal orientation fluctuates over time, no clear positive or negative linear trend over time exists with respect to cadets’ goal orientation in our data. This finding is in line with existing research on goal orientation stability also suggesting relative stability (e.g., Fryer and Elliot, 2007; Corker et al., 2013). Such goal stability can be partly explained by AGT, which suggests that goal orientations are assumed to be relatively enduring self-cognitions (Nicholls, 1984; Roberts, 2012) and that they therefore “may be conceived of as quite stable dispositions for the adoption of certain goal orientations” (Kaplan and Maehr, 2007, p. 164). In support, a more recent study found that only 8.8% teachers change their goal orientation over time (1 year) (Kunst et al., 2018).

Our findings extend prior research by demonstrating that perceived mastery and performance climates explain both within-person and between-person variance in mastery and performance orientations, respectively. This suggests that the perceived motivational climate relates to individual differences in cadets goal orientation as well as changes in their goal orientation over time. Our study thereby contributes to the AGT literature by further clarifying the contextual impact of the perceived motivational climate on goal orientation (cf., Payne et al., 2007; Corker et al., 2013). Specifically, the finding that a perceived mastery climate was clearly predictive of a mastery orientation, while a perceived performance climate predicted performance orientation, supports Ames (1992b,c) theoretical predictions that exposure to a perceived mastery climate encourages a mastery orientation, while a perceived performance climate encourages performance orientation. This underlines the importance of not only studying the relevance of goal orientation (e.g., Corker and Donenellan, 2012) but also how they can develop dynamically in context (Button et al., 1996; DeShon and Gillespie, 2005; Vandewalle et al., 2019). Thus, the decline in mastery orientation over 4 years in Corker et al. (2013) study might be a result of a lack of or decline in perceived mastery climate. If the achievement environment supported the values inherent in a mastery climate, individuals might not experience a drop in mastery goals.

Interestingly, however, whereas the perceived motivational climate explained approximately 50% of the between-person variance in mastery orientation and approximately 14% of the between-person variance in performance orientation, it only explained a small proportion of the within-person variance in mastery (approximately 2%) and performance (approximately 2%) orientations. Accordingly, our findings suggest that perceived mastery and perceived performance climates to a greater extent can explain why cadets differ in mastery and performance orientation as opposed to fluctuations in mastery and performance orientation over time. One possible explanation for this observation is that both the perceived motivational climate and goal orientation were relatively stable across the 2 years of the present study. That is, several aspects of the motivational climate, such as the evaluative structure, instructors’ skill, and frequency of evaluation, also represent stable factors that influence individual goal orientation (Ames, 1992a; Fryer and Elliot, 2007). Hence, given motivational climate stability, goal orientation stability is to be expected.

Our findings support prior theorizing suggesting that goal orientations “not only emerge from stable factors but remain grounded in these factors throughout the process of goal pursuit and regulation” (Fryer and Elliot, 2007, p. 700). Even after the goal has been adopted these antecedents of goal orientation can remain influential (Fryer and Elliot, 2007). In fact, Corker et al. (2013) argued that individual classroom settings are most likely more stable than the broad context of college (or military academy) courses in general. Consequently, such stability most likely facilitates higher stability in goal orientation for specific courses in comparison to goals for courses in general (Corker et al., 2013).

It should also be noted that it seems as if similarity between the motivational climate value system and what the individual values is important. In other words, the mastery-oriented cadet may prefer a mastery climate, while the performance-oriented cadet rather favors a performance climate. This is typically referred to as value congruence and affects a persons’ attitudes and behaviors because people are typically more attracted to and trusting of others who are similar to them (Cable and Edwards, 2004). Thus, when the values of the motivational climate are incongruent with what the person values in terms of his/her goal orientation, it might facilitate negative attitudinal and behavioral outcomes (cf., Cable and Edwards, 2004).

Our results did not indicate a significant negative relationship between a perceived mastery climate and performance orientation as initially hypothesized. A possible explanation for this refusal of our hypothesis may be that it takes longer time than the time frame of this particular study for a mastery climate to reduce or shape cadet’s performance orientation. Another reason may be the criteria of success which are valued in a mastery climate may be perceived to deviate too much from how performance oriented individuals personally define success and how they currently perceive themselves (Wang et al., 2018). Thus, a reduction or change in trait-like related behaviors may not be considered as necessary and/or desirable by the individual, which may explain the refusal of our hypothesis. For a reduction in performance orientation to happen a cadet may have to practice and enact trait-relevant behaviors over time to make them more habitual and in line with what is valued in a mastery climate (cf., Hennecke et al., 2014). Perhaps a focused goal orientation intervention would be needed if the goal is to significantly reduce or even change individual’s performance orientation (cf., Wang et al., 2018). Future research will have to clarify this further.

## Limitations and Future Directions

The methodological strengths as well as limitations of the present research should be acknowledged. The main strength of the present study is that it is based on a longitudinal design. The three-wave longitudinal research design allowed us to establish temporal relationships between the perceived motivational climate and individual goal orientation. Still, our study is not without limitations. First, as with all non-experimental research, we cannot demonstrate causal relations between the variables we studied (Shadish et al., 2001). In addition, there is a potential concern for reverse causality where the possibility exists that goal orientations may drive perceptions of the motivational climate. Experimental studies would be necessary to be able to test and draw causal implications.

Second, our study relies on self-reported data in terms of the measurement of the goal orientations and motivational climate. These are prone to common method bias and inflated ratings in terms of for example the implicit theories of the respondents or social desirability (Podsakoff et al., 2003). However, as individual goal orientation and perceived motivational climate are perceptual variables, the primary interest of our study is clearly best represented by means of self-report. Hopefully, our emphasis on participant confidentiality will have reduced potential common method variance by decreasing the likelihood that respondents “edit their responses to be more socially desirable, lenient, acquiescent, and consistent with how they think the researcher wants them to respond” (Podsakoff et al., 2003, p. 888).

Third, given that our sample consisted of mostly male respondents who were presently enrolled in military academies in Norway, the generalizability of our findings may be limited. A recent meta-analysis from the educational domain did not find significant gender differences in perceptions of the motivational climate or goal orientations, but the world regions of the respondents seem to matter (Bardach et al., 2019). Future research might serve to further clarify the generalizability of our findings across gender, countries/world regions, and cultures.

Fourth, given that we did not measure the cadets’ goal orientation before they entered their studies at the academy, we cannot be certain whether their initial goal orientations in fact have been influenced/changed by the motivational climate in the particular military academy. With our data we may only address whether their goal orientations have remained stable or not during their time of study at the military academies.

Based on our findings and discussion, an interesting avenue for future research could be to investigate the relevance of the strength of the motivational climate for goal orientation stability and/or change (cf., Schneider et al., 2002). Such a research focus might help clarifying the unresolved issues concerning goal orientation variability/stability. For example, Van Yperen et al. (2011) argued the importance of viewing goal orientation as a situational dependent variable because experimental research has shown that individual goal orientation can be successfully manipulated. In addition, Anderman and Midgley (2004) found that students who moved from a low-performance climate to a high-performance climate reported increased cheating behavior. This was also evident for students who moved from a high-mastery climate to a low-mastery climate. Although their study concerned cheating behavior, it addresses the possible impact of a strong climate. These existing findings might indicate that climate strength represents a valuable explaining mechanism in terms of moderating the motivational climate–goal orientation relationship.

## Practical Implications

Our results clearly indicate how a mastery climate is predictive of a mastery orientation, while a performance climate is predictive of a performance orientation. Also, given that the climates were found to explain why individuals differ in mastery and performance orientation rather than why these goal orientations fluctuate over time, our research has important practical implications. First of all, a mastery climate has been found to only predict positive affective, cognitive, and behavioral outcomes, while the opposite is salient for a performance climate (e.g., Ntoumanis and Biddle, 1999; Roberts, 2012). This leads us to argue for the importance of facilitating a mastery climate in achievement contexts, particularly in military academy settings.

In achievement contexts (e.g., military academy, sports, and work), teachers, coaches, and/or leaders’ goals are obvious by how they design achievement sessions, how they evaluate performance, how they give recognition, and by what they perceive as appropriate characteristics (Ames, 1992b). Therefore a teacher/coach/leader can encourage a particular goal orientation by making certain expectations, cues, and rewards salient (Ames, 1992b). This creates the motivational climate and transfers certain goals to individuals (Ames, 1992c). When considering the common application of extrinsic rewards, public evaluation practices, ability grouping, normative comparisons, and emphasis on perfection, production, and speed in various achievement contexts, it is no surprise that individuals find it hard to maintain their mastery orientation (Ames, 1990). Thus, in military academies (and other settings), leaders and teachers may be well-advised to reflect upon the approaches they use and signals they send in terms of individual behavior they value, expect, and reward. Such signals are likely to affect cadets’ climate perceptions, motivation, attitudes and retention (Langkamer and Ervin, 2008; Magnussen and Boe, 2017).

To facilitate a mastery climate and consequently foster mastery orientation, the emphasis should be on the value of learning, development and cooperation (e.g., Chen and Mathieu, 2008; Van Yperen et al., 2011). One such approach is the task, authority, recognition, grouping, evaluation, and time (TARGET) framework (Epstein, 1989; Ames, 1992b). In line with this framework, *tasks* should be meaningful and involve novelty, diversity, variety, problem solving, and/or discovery (Ames, 1992c; Valentini and Rudisill, 2006). Individuals should also receive the *authority* to participate in decision making, receive encouragement to initiate activities, and make task choices to support their autonomy (Valentini and Rudisill, 2006; Roberts, 2012). Further it is important to *recognize* and reward individual improvement and progress privately (and not in comparison to significant others) (Roberts, 2012). Such an approach respects and enhances individuals’ sense of self-worth (Ames, 1992c). The *grouping* dimension refers to the value of diversity, acceptance of individual differences, and the importance of strengthening feelings of belongingness (Valentini and Rudisill, 2006). Belongingness is fostered through cooperative work and the encouragement of information sharing to help each other in problem solving. The provision of an effective *evaluation* system where individuals’ efforts and improvements are privately and not publicly acknowledged is also important (Valentini and Rudisill, 2006; Roberts, 2012). In line with Nicholls (1989), public evaluation only provokes concern regarding the adequacy of an individual’s competence and consequently increases the tendency to consider competence as capacity (Valentini and Rudisill, 2006). Lastly, the *time* dimension of TARGET refers to the pace of instruction, workload adequacy, and learning tasks time. In a mastery climate opportunities and time for improvement are provided (Valentini and Rudisill, 2006). Some individuals’ might need more time to develop their potential.

## Data Availability Statement

The datasets presented in this article are not readily available because of the regulations set by the Norwegian Center for Research Data. This is to protect respondent confidentiality and participant privacy. Requests to access the dataset should be directed to RS, reidar.safvenbom@nih.no.

## Ethics Statement

Ethical review and approval was not required for the study on human participants in accordance with the local legislation and institutional requirements. The participants provided their written informed consent to participate in this study.

## Author Contributions

CN, RB, AD, and RS contributed to the conception and design of the study. RS organized the database. RB performed the statistical analysis and wrote sections of the manuscript. CN wrote the first draft of the manuscript. All authors contributed to manuscript revision, read and approved the submitted version.

## Conflict of Interest

The authors declare that the research was conducted in the absence of any commercial or financial relationships that could be construed as a potential conflict of interest.
